# Magnetic fields from skeletal muscles: a valuable physiological measurement?

**DOI:** 10.3389/fphys.2015.00228

**Published:** 2015-08-10

**Authors:** Marco A. C. Garcia, Oswaldo Baffa

**Affiliations:** ^1^Departamento de Biociências da Atividade Física, Escola de Educação Física e Desportos, Universidade Federal do Rio de JaneiroRio de Janeiro, Brasil; ^2^Departamento de Física, Faculdade de Filosofia, Ciências e Letras de Ribeirão Preto, Universidade de São PauloRibeirão Preto, Brasil

**Keywords:** biomagnetic measurements, *magnetomyography*, MMG, *magnetomyogram*, skeletal muscle, MMG signal, magnetometer

It is well known that whenever a motor unit is recruited, electrical, and mechanical events are generated in skeletal muscle fibers. The first concerns to the action potentials propagating along the sarcolemma while the second is represented by dimensional changes that are interpreted as mechanical twitches. Both allow the regulation of muscle force production. Electrical and mechanical events can be indirectly measured by means of different transducers that can provide details concerning the underlying mechanisms of muscle contraction elicited under different conditions. Therefore, surface or indwelling electrodes, laser distance sensors, microphones, and accelerometers are examples of transducers commonly used by those who are interested in interpreting how muscles control joints and regulate force production, among other properties. Thus, electromyography (EMG) and mechano or vibro or acceleromyography (usually named MMG) are examples of well-established methods that are widely used today in basic, sports and clinical studies. However, another physical quantity, magnetic fields associated with the flux of ions across the active cells membranes, has been well reported in organs such as brain (Hari and Salmelin, 1997, 2012; Nevalainen et al., 2014) and heart (Geselowitz, 1979; Fenici et al., 2005; Leithäuser et al., 2011) although much less frequently for the skeletal muscles. Interestingly, though, few previous authors have pointed out that the recording of magnetic fields of this tissue may help to improve our knowledge in respect of its physiology under normal and pathological conditions. As an example of these pioneers, we may mention the study conducted by Van Egeraat et al. (1990). They carried out the first recordings of magnetic fields generated by a single skeletal muscle fiber and provided details concerning some cellular properties such as membrane capacitance and intracellular conductivity, which are notoriously helpful in basic physiology. Moreover, some authors also support the idea that measuring magnetic fields from this tissue can provide additional details concerning muscle gradation force mechanisms (Lewis, 2003), which is of utmost importance in clinical and sports applications. Thus, why so few studies? What are the constraints, disadvantages and advantages of measuring magnetic fields from skeletal muscles? May it provide valuable and feasible data for a better understanding of skeletal muscle physiology?

It is interestingly to note that due to the source of the phenomenon, some authors have referred to this approach as *Magnetomyography* (also abbreviated as MMG and from now on in the present text not referred to the mechanical events derived from muscle contraction) and the raw signal as *magnetomyogram* (Cohen and Givler, 1972; Rutten et al., 1989; Wikswo et al., 1989; Wijesinghe, 2014). MMG is based on the measurement, usually not invasively (few studies were conducted in isolated muscle fibers, as can also be seen in Van Egeraat et al., 1990), of the magnetic field generated by the skeletal muscle fibers under contraction. The earliest study in MMG was conducted more than 40 years ago by Cohen and Givler and firstly defined by these authors as being the “*recording of one component of the magnetic field vector vs. time, where the magnetic field at the point of measurement is due to currents generated by skeletal muscle.*” In summary, an ionic current flowing in a muscle fiber will generate concentric and circular magnetic fields that can be detected on the skin surface by a couple of transducers whose basic mechanism is given by the Biot Savart law and that its direction is also easily described by the *right hand rule*. Another important characteristic of the MMG signal is that it represents a linear summation of those magnetic fields generated by each depolarized skeletal muscle fiber that, in turn, is dependent upon muscle architecture (Wijesinghe, 1991). Therefore, alternatively to the methods previously mentioned, MMG aims to interpret skeletal muscle contraction mechanisms from the magnetic fields produced by the same ionic currents that give rise to the EMG signal (Cohen and Givler, 1972; Parker and Wikswo, 1997). It is also important to note that other studies also refer to MMG to the monitoring of uterine contraction during pregnancy. Interesting details about this particular application can be seen elsewhere (Nagarajan et al., 2003; Govindan et al., 2015).

The magnitude of the MMG signal is lower when compared to other biological tissues (heart, for example) and can range from pico (10^−12^) to femto (10^−15^) Tesla (T), depending on the approach of measurement. In this respect, the MMG signal can be collected by means of toroidal pickup coils and Superconducting Quantum Interference Devices (SQUIDs). Wound toroidal pickup coils can have different sizes of cores, wire gauges and number of turns (Williamson et al., 1983) (Figure 1A). As action potentials propagate along skeletal muscle fibers, the magnetic fields induced around them will induce a current in the toroid by Faraday's Law (Skoczelas, 2009). In turn, SQUIDs are characterized by an array of one or more coils immersed in liquid helium to maintain a superconductive state (Figure 1B). Although both magnetometers can be used in MMG signal acquisitions, SQUIDs allow detecting such low range of magnetic fields with a higher spatial resolution in comparison to wound toroidal pickup coils and have been preferably adopted by most of authors whenever possible.

**Figure 1:**
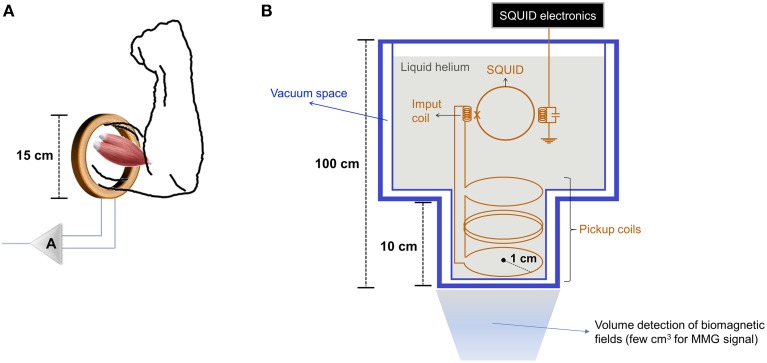
**Drawings of basic designs of (A) a toroidal pickup coil built for collecting MMG signals from biceps *brachii muscle* (adapted from Nantel and Pengelly, 1991; A: Amplifier system), (B) a SQUID (one channel) set-up mounted in an insulating vessel (adapted from Oschman, 2002)**. The measures given are approximate guides.

The MMG signal is biphasic in shape and seems to present a spectral content quite similar to the EMG signal collected from surface electrodes (Cohen and Givler, 1972). However, although both may appear morphologically similar, unlikely the EMG, the MMG is potentially superior in providing additional details concerning skeletal muscle contraction mechanisms in contrast to the previous one. We support this hypothesis due to the fact that electrical fields are disrupted by the various layers of tissue between the source and the skin surface besides the dc and noise voltages originated at the skin-electrode interface (Merletti and Parker, 2004; Cavalcanti Garcia and Vieira, 2011), whereas these tissues are essentially a “way open” to magnetic fields (Oschman, 2002). Therefore, detecting skeletal muscle activity with magnetometers without any physical contact eliminates the possibility of disrupting the MMG signal in the same way we can usually experience in typical EMG signal acquisition.

Among the clinical and research applications of MMG, it has been shown that this approach allows localizing the current source in a skeletal muscle. It seems possible due to an apparent difference in amplitude of the MMG signal collected near from the innervation zones in comparison to other muscle spots and without any physical contact between the transducer and the skin as just mentioned (Koga and Nakamura, 1983; Oschman, 2002; Wijesinghe, 2014). It sounds interesting, for instance, in localizing motor points in *botulinum* toxin applications (Guzmán-Venegas et al., 2014) and peripheral electrical stimulation (Gobbo et al., 2014), which is clinically relevant in both cases although high density surface EMG has been shown as robust alternative in this concern (Cavalcanti Garcia and Vieira, 2011; Barbero et al., 2012). In addition, when skeletal muscle fibers are injured, there is a leakage of current that can be easily detected by SQUIDs, which also help to better map sites of muscle lesions (Curio et al., 1993; Mackert et al., 1999). Another important issue is that the MMG signal amplitude seems to be linearly correlated with muscle force production (Rutten et al., 1989; Gielen et al., 1991) in contrast to the EMG signal that is still under discussion (Hashemi et al., 2015). It can be especially useful in the simplification of mathematical models with added reliability in predicting muscle force production from the MMG signal instead the EMG.

Even though there is clear evidence of the potential use of the MMG in skeletal muscle contraction analysis, there seems to also be methodological and cost constraints that still restrict a larger use and corroborate the lack of studies. For instance, considering that the earth's magnetic fields can reach values of the order of 10^−6^ T and the typical magnetic urban noise can be on the order of 10^−7^ T/(Hz)^1∕2^, collecting the MMG signal with wound toroidal pickup coils may lead to the need of performing experiments in shielded rooms to avoid this larger background field (Lewis, 2003). Albeit Nantel and Pengelly (1990); Nantel and Pengelly (1991) reported the development of a wound toroidal pickup coil constituted by additional elements (ex: thin copper-beryllium foil) and a method of digital filtering, both to improve the MMG signal noise ratio, no progress seems to be performed. Moreover, MMG signal amplitude varies with the third power of the distance between the transducer and the current source. As a result, significant dimensional changes of the skeletal muscle during contraction or a movement of the volunteer or the body part under investigation can change the MMG signal, which can be troublesome. Consequently, all the human studies *in vivo* collected the MMG signal while volunteers performed isometric contractions (Cohen and Givler, 1972; Koga and Nakamura, 1983; Masuda et al., 1999). Furthermore, as a superconductor device, SQUIDs require to be cooled with liquid helium, which is operationally difficult to handle and very expensive, limiting a wide use of this approach. Alternatively, few authors have also attempted to detect magnetic fields of neural and skeletal muscle fibers using magnetic resonance imaging (MRI) although they did not succeed yet (Wijesinghe and Roth, 2009; Roth et al., 2014). The authors report that to achieve progress in such approach, there must be necessary to overcome some technical constraints. Among them, we may cite the magnetic susceptibility inhomogeneities, i.e., the degree of magnetization of skeletal muscles in response to an applied magnetic field. The skeletal muscles present a magnetic susceptibility lower when compared to other biological tissues and organs (ex: brain, heart, bones, among others), which constitutes a limiting factor in MRI as an adjuvant approach. In turn, some authors (Truong and Song, 2006; Roth and Basser, 2009; Wijesinghe and Roth, 2009; Roth et al., 2014) have argued to record, mainly the neural activity, based on Lorentz effect. The Lorentz effect relies on the idea of a conductor carrying a current exposed to a magnetic field can be submitted to a movement enough to cause a disturbance in the spin dynamics, which would result in an artifact in the MRI signal. However, these authors also suggest that such movement is too small to be detected even by the currently MRI technology. Nevertheless, we must emphasize that most of trials were conducted with neural tissue, which must be seen with caveats since the literature provide few studies particularly with respect to skeletal muscles and even though Wijesinghe and Roth (2009) suggested that recording magnetic signals of these tissues is still far from “reality.”

Notwithstanding, we must assume that further progress needs to be made toward to improving the methodological approach in recording magnetic fields of skeletal muscles, we may expect that MMG will be beneficiated by recently technological advances. For instance, there is ongoing research to develop high sensitive magnetometers that do not need cryogenics that can modify this scenario. In the last decades, the atomic magnetometers have reached higher levels of sensitivity (subfemtotesla), more compact designs, with few cubic centimeters, and with no need of being cryogenically cooled (Kominis et al., 2003; Shah and Wakai, 2013). In addition, ultra-low-field (ULF) MRI has also been pointed out as an alternative to typical MRI systems to recording magnetic fields of neural tissues, which may be applied to skeletal muscle tissues (Wijesinghe and Roth, 2009). In contrast to typical MRI systems, ULF MRI operates at μT fields (Kraus et al., 2008), which would allow overcoming the constraints imposed by the magnetic susceptibility in recording magnetic fields of skeletal muscles by this technology.

In the light of the aforementioned, can magnetic fields from skeletal muscles become a valuable physiological measurement? Indeed, we may say *yes*. Many technological advances have been made and, as a result, different approaches have emerged as alternatives to other magnetometers such as SQUIDs in biomagnetic measurements. Therefore, we conclude that, MMG may become in the near future a promising and complementary approach to electrical and mechanical recordings in skeletal muscle physiology studies.

## Conflict of interest statement

The authors declare that the research was conducted in the absence of any commercial or financial relationships that could be construed as a potential conflict of interest.
